# Functional neurological symptoms are a frequent and relevant comorbidity in patients with multiple sclerosis

**DOI:** 10.3389/fneur.2023.1077838

**Published:** 2023-04-11

**Authors:** Katya Piliavska, Michael Dantlgraber, Christian Dettmers, Michael Jöbges, Joachim Liepert, Roger Schmidt

**Affiliations:** ^1^Lurija Institute for Rehabilitation Sciences and Health Research, Allensbach, Germany; ^2^Kalaidos University of Applied Sciences, Zürich, Switzerland; ^3^Kliniken Schmieder Konstanz, Konstanz, Germany; ^4^Kliniken Schmieder Allensbach, Allensbach, Germany; ^5^Klinik für Psychosomatik und Konsiliarpsychiatrie, Kantonsspital, St. Gallen, Switzerland

**Keywords:** functional neurological symptoms, multiple sclerosis, health-related quality of life, work ability, neurological rehabilitation

## Abstract

**Introduction:**

Functional neurological symptoms (FNS) in multiple sclerosis (MS) have shown to be underinvestigated even though neurological diseases such as MS represent a risk factor for developing FNS. Comorbidity of FNS and MS can produce high personal and social costs since FNS patients have high healthcare utilization costs and a quality of life at least as impaired as in patients with disorders with underlying structural pathology. This study aims to assess comorbid FNS in patients with MS (pwMS) and investigate whether FNS in pwMS are associated with poorer health-related quality of life and work ability.

**Methods:**

Newly admitted patients (234) with MS were studied during their stay at Kliniken Schmieder, a neurological rehabilitation clinic in Konstanz, Germany. The degree to which the overall clinical picture was explained by MS pathology was rated by neurologists and allied health practitioners on a five-point Likert scale. Additionally, neurologists rated each symptom reported by the patients. Health-related quality of life was assessed using a self-report questionnaire and work ability was assessed using the mean number of hours worked per day and information regarding disability pension as reported by patients.

**Results:**

In 55.1% of cases, the clinical picture was completely explained by structural pathology due to MS. 17.1% of pwMS presented an overall clinical picture half or less of which could be explained by underlying structural pathology. PwMS with a higher comorbid FNS burden had a lower health-related quality of life and reported fewer working hours per day than pwMS with symptoms explained by structural pathology. Furthermore, pwMS with a full disability pension had a higher comorbid FNS burden than pwMS with no or partial disability pension.

**Discussion:**

These results show that FNS should be addressed diagnostically and therapeutically since such symptoms are an important comorbidity in MS that is related to poorer health-related quality of life and lower work ability.

## 1. Introduction

About 30% of neurological patients have symptoms that are only partially or not at all explained by underlying structural pathology (1). These so-called functional neurological symptoms (FNS) are among the most common causes of disability in neurology (2). Patients with definite structural neurological pathology often demonstrate FNS (20%) (3), and the presence of a neurological disease is even considered a powerful risk factor for developing FNS (4). Although FNS are a frequent comorbidity in diseases with an underlying structural pathology such as multiple sclerosis (MS), empirical findings on the prevalence of such symptoms in MS are quite scarce. One recent study found that MS appears to be less associated with FNS than with migraine, cerebrovascular diseases, and Parkinson’s Disease (5). Our first investigation on functional somatic symptoms in MS demonstrated that about 60% of patients showed symptoms that could not be entirely explained by underlying MS pathology (6). The term “functional somatic symptoms” includes a broad spectrum of neurological as well as gastrointestinal, cardiovascular, and pain symptoms with clinical findings inconsistent with structural pathology. Prior research conducted mostly as case studies suggested a tendency to functional symptoms (7), which might even appear as pseudo relapses in MS (8, 9). As for clinical practice, this comorbidity is often overlooked or neglected by doctors. It is a common misconception of clinicians that there is no need to look for further explanations in the presence of a disease with an underlying structural pathology (10). Moreover, some neurologists view FNS not as a neurological problem or disease and consider such symptoms as a psychiatrist’s responsibility (11).

Assessing comorbid FNS in MS can be very challenging and requires thorough neurological examination and comprehensive clinical history taking. The diagnosis should always be made based on positive evidence. As for neurological examination, inconsistency (i.e., changing patterns over time with susceptibility to distraction) and/or incongruence (i.e., a clinical picture incompatible with known organically determined patterns) of clinical findings are crucial in diagnosing such symptoms. Diagnostic process can be supplemented by an independent medical history-taking and functional diagnosis by an allied health practitioner, i.e., physiotherapist or occupational therapist (2). Our previous study suggests that assessing psychopathology in patients with MS (pwMS) might be helpful in evaluating possible comorbidity with FNS since pwMS with more functional somatic symptoms showed higher rates of depression, anxiety and dissociation than pwMS with symptoms completely explained by MS pathology (6). Clinical experience suggests an integrated multidisciplinary approach to be very helpful in diagnosis (and treatment) of comorbid FNS, as it includes assessment by a multi-professional team of physicians, nurses, occupational and physiotherapists, (neuro)psychologists and psychotherapists. For research purposes, comorbid FNS can be assessed by a physician and allied health practitioner using “organicity ratings.” Likert-scales are used to assess the extent of the overall clinical picture explained by underlying structural pathology (3, 12, 13). In our study, we omit the use of the terms “organic” and “organicity” since the “organic” vs. “non-organic” distinction is outdated as it promotes dualistic thinking.

One should pay attention to the possible comorbidity of FNS in MS since both conditions carry high personal and social costs. FNS does not only cause physical disability comparable with other structural neurological conditions like epilepsy, FNS patients are burdened by more psychological comorbidities than patients with neurological disorders (14) and have a quality of life at least as altered as patients with disorders with underlying structural pathology (15). FNS patients have high healthcare utilization costs related to clinic visits, unnecessary diagnostic procedures, and treatments with healthcare utilization costs comparable to patients with care-intensive neurological conditions (16). Therefore, an early and correct diagnosis of comorbid FNS plays a crucial role since delayed diagnosis and long symptom duration negatively affect the prognosis (17).

Since comorbid FNS potentially pose a serious complication in addition to MS, this study aimed to investigate the prevalence of FNS in MS and to replicate our previous results (6). Moreover, we examined whether health-related quality of life and work ability differ between pwMS with higher vs. lower FNS burden. We use the term “functional neurological symptoms” more broadly in this paper by including symptoms such as pain, fatigue, etc. that are beyond the diagnostic criteria defined in the fifth edition of the Diagnostic and Statistical Manual of Mental Disorders (DSM-5).

## 2. Methods

The study was approved by the Ethics Committee of the University of Konstanz and was conducted according to the declaration of Helsinki.

### 2.1. Study design and procedure

Data were collected between August 2020 and October 2021 at Kliniken Schmieder in Konstanz, Germany. Kliniken Schmieder is a neurological rehabilitation and specialist clinic for neurology with patients being admitted both directly from acute care hospitals and by referral from neurologists. During an average stay of 5 weeks, patients are treated by a multidisciplinary team of physicians, nurses, occupational and physiotherapists, (neuro)psychologists and psychotherapists.

**Figure 1:**
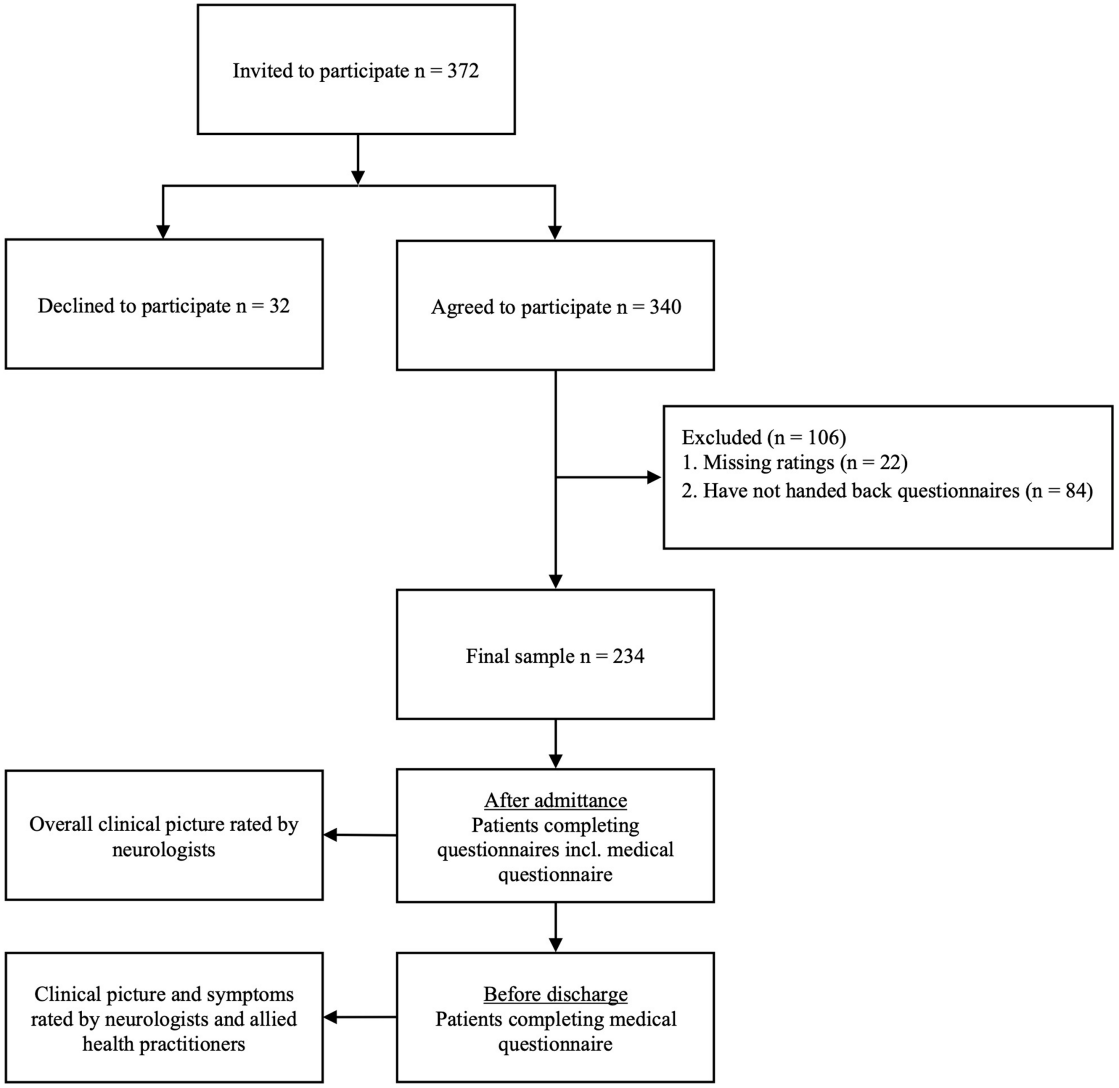
Flowchart illustrating study design.

The sample included newly admitted patients between 18 and 60 years with definite MS diagnosis and a prospective duration of treatment of at least 3 weeks (*n* = 234; Figure 1). Exclusion criteria were motor or cognitive deficits as a consequence of another neurological disorder (i.e., stroke) and motor or cognitive deficits that make it impossible to fill out the questionnaires. Patients who developed acute relapses during the course of their stay were excluded from the study.

The study was presented individually to every eligible pwMS after admittance. After providing consent, patients received a set of questionnaires including a medical questionnaire assessing neurological symptoms in the last 3 months, and were asked to fill them out during the first week of their stay. In addition, a medical questionnaire assessing symptoms was handed out in the last 7 days shortly before discharge. Senior neurologists rated the overall clinical picture after admittance. The symptoms in the medical questionnaire reported shortly before discharge as well as the overall clinical picture were rated by senior neurologists and allied health practitioners (occupational or physiotherapists) independently of each other (Figure 1). Symptom evaluation after admittance and before discharge was conducted by the same neurologist. Neurological assessments and ratings have been conducted in the inpatient setting at Kliniken Schmieder.

### 2.2. Materials

Neurological symptoms were assessed with an adapted medical questionnaire from our previous study (6). It included 18 neurological symptoms: memory disturbances, problems with swallowing, sexual dysfunction, speech problems, cognitive fatigue, motor fatigue, pain, tremor, muscular twitching/cramps, increased muscular tonus, paralysis/muscular weakness, visual impairments, sensory disturbances, coordination/balance disorders, gait disorders, fine motor skills disorders, difficulty in/too frequent urination, epileptic seizures.

We have included a broader spectrum of functional neurological symptoms (e.g., pain, fatigue) that are beyond the diagnostic criteria defined in the DSM-5. DSM-5 criteria for diagnosing functional neurological disorder refer to altered voluntary motor and/or sensory functions only. Symptoms like pain, cognitive dysfunction or fatigue can fluctuate in a way comparable to FNS. By including these symptoms, we try to take into account the reality of everyday clinical practice where such symptoms are often present.

The degree to which each reported symptom and the overall clinical picture was explained by underlying structural pathology due to MS was rated by senior neurologists and allied health practitioners on a five-point Likert scale. The options on the scale to select were as follows: 1—not at all explained by underlying structural pathology, 2—partly, 3—about a half, 4—predominantly, and 5—completely explained by underlying structural pathology. A higher numerical score, therefore, represented a higher extent of symptoms explained by underlying structural pathology.

The assessment was based on the information from clinical examination, personal and third-party medical history and further findings (i.e., electrophysiological tests). Neurologists and allied health practitioners were trained in rating the symptoms before the study using positive or “rule-in” neurological examination signs. Inconsistency (i.e., changing patterns over time with susceptibility to distraction) and/or incongruence (i.e., a clinical picture incompatible with known organically determined patterns) of clinical findings were crucial diagnostic aspects in rating the symptoms. Inconsistency and incongruence were assessed not exclusively by the presence of “rule-in” examination signs, but also by considering other aspects such as distractibility and fluctuations of the symptoms. The treatment context of neurological rehabilitation offers plenty of different and recurring situations in which patients and their symptoms can be observed. For example, change of symptoms when a patient feels observed in the therapy sessions vs. being in a familiar and relaxed situation with friends or family.

Health-related quality of life (HRQoL) was assessed with the German version of the Short Form 36-Item Health Survey (SF-36) consisting of 36 items (18). SF-36 comprises eight subscales (physical functioning, physical role limitations, bodily pain; general health perceptions; vitality, social role functioning, emotional role functioning, mental health/emotional well-being). Higher scores represent better HRQoL. Two summary dimensions can be obtained from the abovementioned subscales: physical health score and mental health score.

Work ability was measured by the mean number of hours worked per day as reported by patients. Moreover, information regarding disability pension was used, as it is a more objective measure of work ability. Patients were asked whether they receive a disability pension with answer options: no disability pension, partial disability pension, or full disability pension.

### 2.3. Statistics

Statistical analyses were performed using SPSS (Version 28; SPSS Inc., Chicago, Illinois). Two-tailed t-test for independent samples was used to compare pwMS with higher FNS burden (overall clinical picture rating of < 4) vs. pwMS with symptoms explained by structural pathology due to MS (overall clinical picture rating of ≥ 4) in regards to HRQoL (SF-36 subscales) and work ability. Bonferroni correction was applied to correct for multiple comparisons. To obtain more balanced group sizes the cut-off value of ≥ 4 was chosen. Levene’s test was used to test for homogeneity of variance. Two-tailed t-tests for independent samples were used to compare pwMS with no disability pension vs. pwMS with partial disability pension vs. pwMS with full disability pension.

## 3. Results

Table 1 provides an overview of the demographical and clinical data of the sample.

**Table 1:** Demographical and clinical data (*N* = 234).

Age in years, *M* (*SD*)	48.7	(8.95)
Women, *n* (%)	168	(71.8)
Family status, *n* (%)		
Married	135	(57.7)
Not married, in a relationship	5	(2.1)
Single	61	(26.1)
Divorced	30	(12.8)
Widowed	3	(1.3)
Years since first manifestation, *M* (*SD*)	15.9	(9.96)
Years since diagnosis, *M* (*SD*)	13.5	(9.03)
EDSS-score, *M* (*SD*)	3.69	(1.56)
MS course, *n* (%)		
Relapsing–remitting	154	(66.1)
Primary progressive	30	(12.9)
Secondary progressive	50	(21)

EDSS = Expanded Disability Status Scale.

### 3.1. Prevalence of comorbid functional neurological symptoms (FNS)

**Figure 2:**
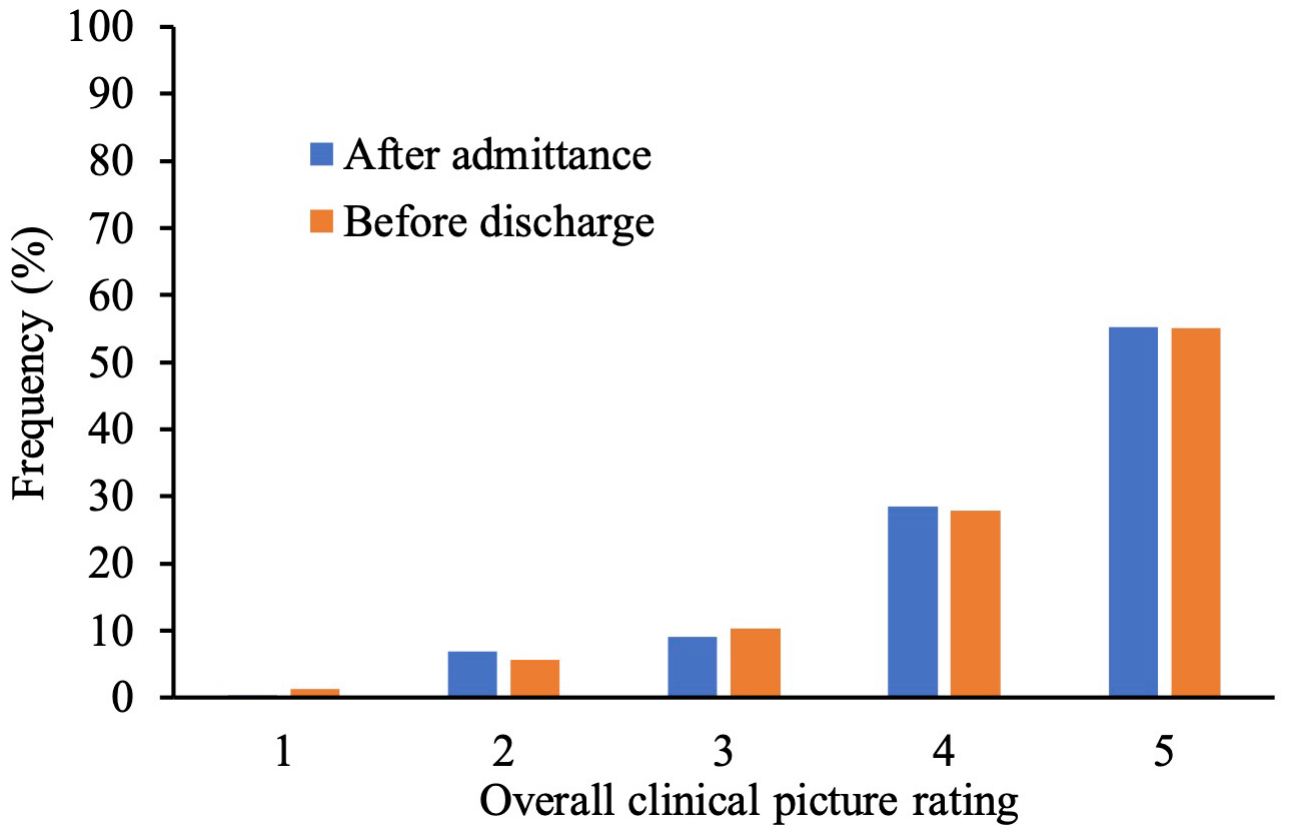
Bar graph Illustrating frequencies (%) of rating (1–5) given by neurologists at the beginning and at the end of patient’s stay. A higher numerical score represents higher extent of system explained by underlying structural pathology.

Figure 2 demonstrates the frequency distribution of neurologists’ ratings after admittance and before discharge. These ratings were found to be positively correlated, *r* = 0.78, *p* < 0.001.

Regarding ratings at the end of the patients’ stay 1.3% of pwMS had an overall clinical picture not at all explained by underlying structural pathology due to MS (rating of 1) and in 5.6% of the cases it was partly explained (2). In 10.3% of pwMS, the clinical picture was half explained by structural pathology (3). 27.8% of the sample showed an overall clinical picture predominantly explained by structural pathology (4) whereas in 55.1% of pwMS, it was completely explained (5).

**Figure 3:**
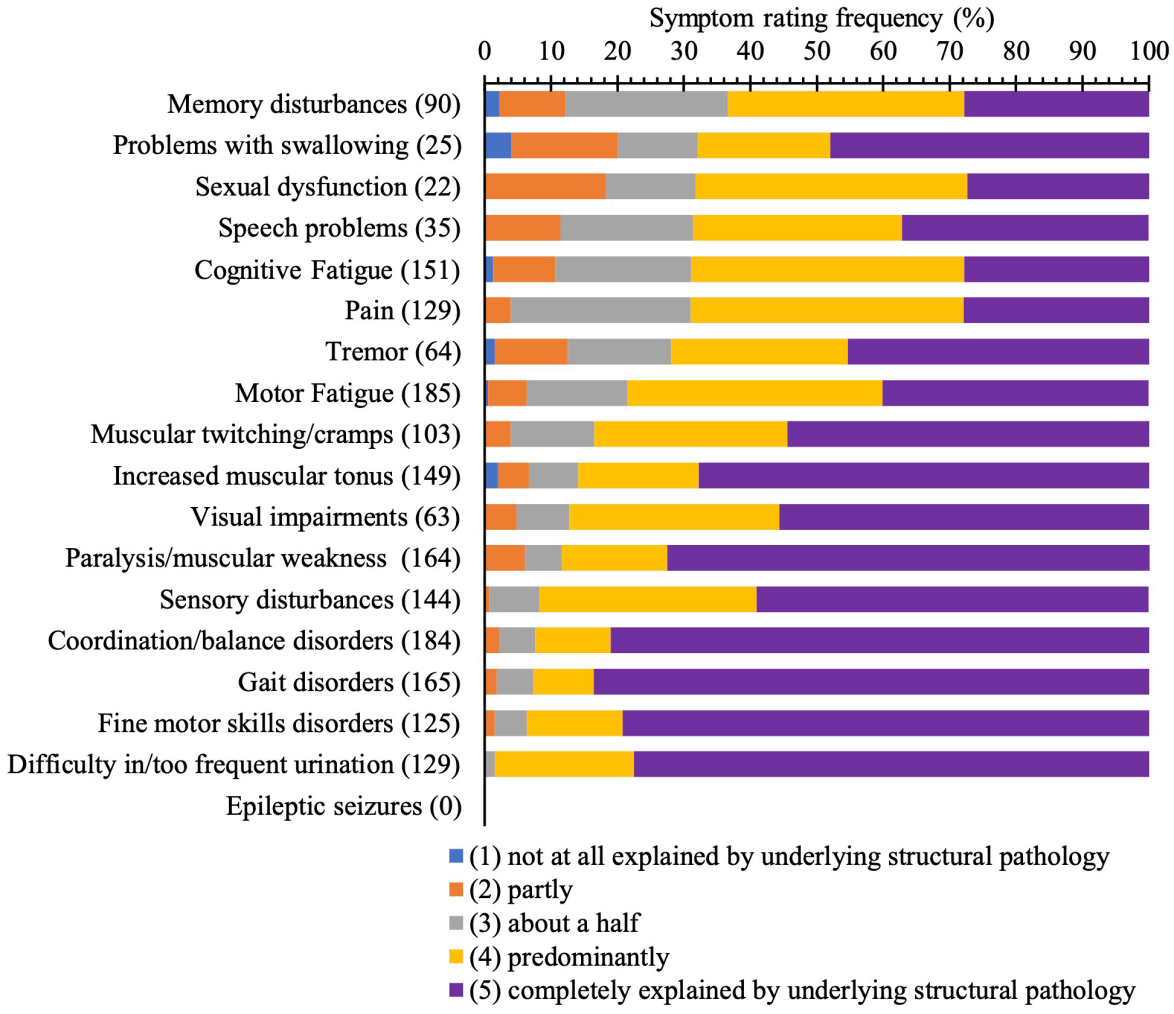
Bar graph illustrating frequencies of neurologist’s rating of each symptom (%, in color) Absolute frequencies of symptoms are indicated in brackets.

Figure 3 depicts the absolute frequency of symptoms (in brackets) in the sample of 234 patients and the frequency distribution of symptom ratings provided by neurologists.

**Figure 4:**
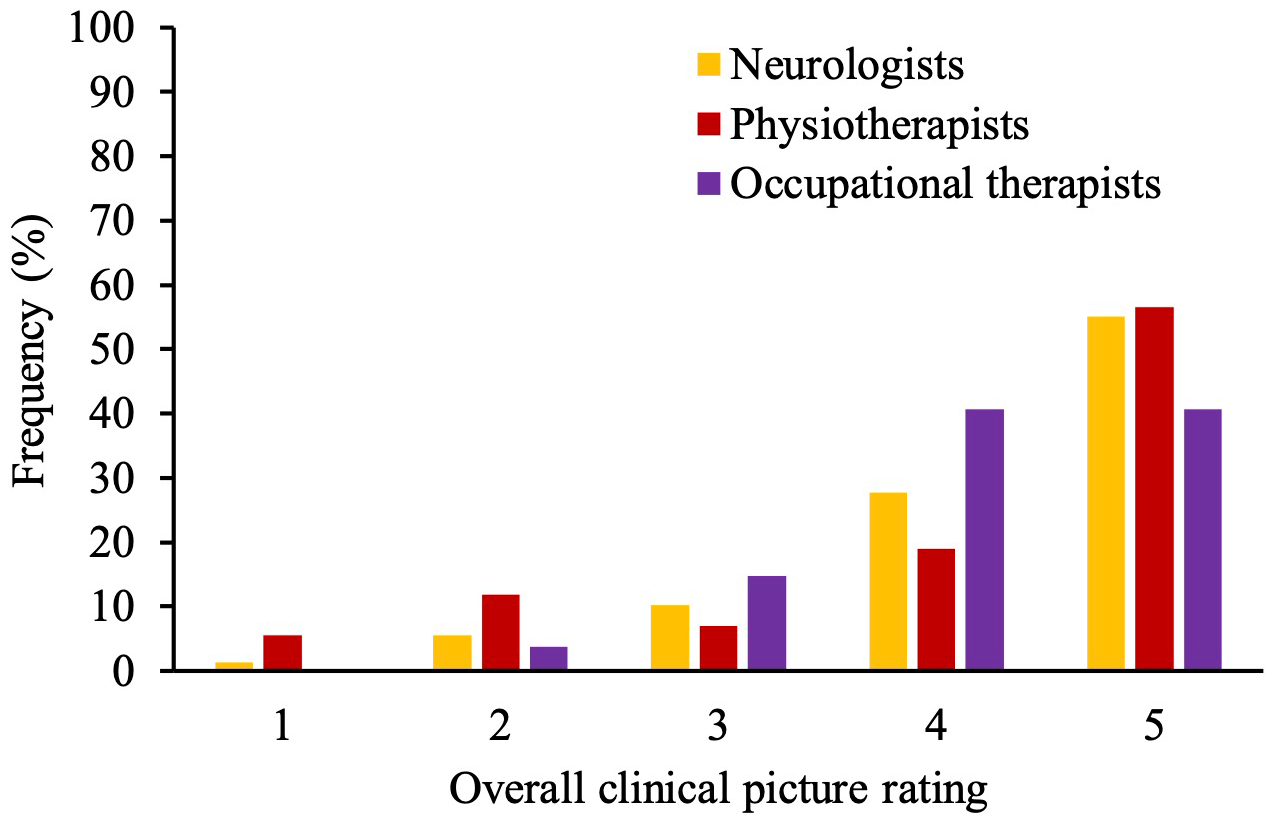
Bar graph demonstrates frequencies (%) of rating (1–5) given by neurologists, physio- and occupational-therapists at the end of patient’s stay. A higher numerical score represents higher extent of symptoms explained by underlying structure pathology.

Figure 4 shows the frequency distribution of overall clinical picture ratings conducted by neurologists, physio- and occupational-therapists before discharge. Neurologists and physiotherapists showed a higher correlation in their ratings (*r* = 0.67, *p* < 0.001) whereas occupational therapists’ ratings correlated poorly with neurologists’ (*r* = −0.02, *p* = 0.91) and physiotherapists’ ratings (*r* = 0.31, *p* = 0.13).

### 3.2. Work ability and health-related quality of life in pwMS with vs. without comorbid FNS

PwMS with higher comorbid FNS burden (ratings of < 4) reported significantly fewer working hours per day than pwMS with symptoms explained by structural pathology due to MS (ratings of ≥ 4) corresponding to a medium effect size (Cohens’ *d* = 0.52). Significant group differences were also found with regard to all SF-36 subscales with pwMS with higher comorbid FNS burden showing lower HRQoL on all subscales except “Physical functioning” (for more details see Table 2).

**Table 2:** Comparison of mean working hours per day and mean SF-36 subscales scores between patients with multiple sclerosis (pwMS) with more functional neurological symptoms (FNS; < 4) vs. pwMS with more symptoms explained by structural pathology (≥ 4).

	< 4 (*n* = 40)	≥ 4 (*n* = 194)	*p*	*d*
	*M (SD)*	*M (SD)*		
Working hours per day	4.64 (3.4)	6.12 (2.7)	**0.015**	0.52
SF-36				
Physical functioning	59.75 (31.09)	48.73 (28.59)	**0.03**	−0.38
Role physical	31.25 (35.69)	45.87 (40.58)	**0.035**	0.36
Bodily pain	47.72 (34.89)	61.84 (29.61)	**0.008**	0.46
General health	34.77 (17.78)	44.40 (19.42)	**0.004**	0.5
Vitality	26.25 (15.67)	38.06 (19.66)	**<0.001**	0.62
Social functioning	37.18 (23.76)	56.76 (25.67)	**<0.001**	0.77
Role emotional	30.00 (39.80)	62.02 (42.73)	**<0.001**	0.75
Mental health	45.20 (20.31)	61.03 (20.67)	**<0.001**	0.76

SF-36 = Short Form 36-Item Health Survey; higher scores represent better health related quality of life Significant differences are indicated in bold. Underlined differences are still significant after using the Bonferroni correction. *p* = value of *p* of *t*-tests for independent samples; *d* = Cohens’ *d*.

Patients with multiple sclerosis with full disability pension had lower overall clinical picture ratings than pwMS with no disability pension (Table 3) and partial disability pension (Table 4). There was no significant difference between pwMS with no disability pension and pwMS with partial disability pension [*t*(221) = −0.39, *p* = 0.69].

**Table 3:** Comparison of mean overall clinical picture ratings between pwMS who do not receive disability pension vs. pwMS who receive full disability pension.

	No disability pension (*N* = 179)	Full disability pension (*N* = 10)	*p*	*d*
	*M (SD)*	*M (SD)*		
Overall clinical picture rating	4.32 (0.97)	3.6 (1.07)	**0.023**	0.74

A higher numerical score of overall clinical picture rating represents higher extent of symptoms explained by underlying structural pathology due to MS.

Significant differences are indicated in bold. *p* = value of *p* of *t*-tests for independent samples; *d* = Cohens’ *d*.

**Table 4:** Comparison of mean overall clinical picture ratings between pwMS who receive partial disability pension vs. pwMS who receive full disability pension.

	Partial disability pension (*N* = 44)	Full disability pension (*N* = 10)	*p*	*d*
	*M (SD)*	*M (SD)*		
Overall clinical picture rating	4.39 (0.78)	3.6 (1.07)	**0.01**	0.93

A higher numerical score of overall clinical picture rating represents higher extent of symptoms explained by underlying structural pathology due to MS.

Significant differences are indicated in bold. *p* = value of *p* of *t*-tests for independent samples; *d* = Cohens’ *d*.

## 4. Discussion

Only 55.1% of cases had a clinical picture that is completely explained by structural pathology due to MS. 17.1% of pwMS presented an overall clinical picture half or less of which could be explained by underlying structural pathology. Neurologists showed high consistency in rating the overall clinical picture at the beginning and the end of a patient’s stay. Memory disturbances (36.6%), problems with swallowing (32%), sexual dysfunction (31.8%), speech problems (31.4%), pain (31%), and tremor (28.1%) were most often rated as half or less explained by structural pathology. Gait (83.6%) and coordination/balance disorders (81%), fine motor skills disorders (79.2%), and difficulty in/too frequent urination (77.5%) were most often thought to be completely explained by underlying structural pathology. These findings may reflect that neurologists’ diagnostic certainty is higher in the presence of secure clinical signs.

Neurologists, physio- and occupational-therapists seem to differ in their ratings which could be because allied health practitioners diagnose and treat only certain symptoms whereas neurologists see the whole clinical picture and take into account findings from other diagnostic procedures (i.e., electrophysiology). On the other hand, allied health practitioners see patients more often than neurologists. They observe patients during different activities of daily living or notice inconsistencies when patients feel observed. Therefore, they might have a more accurate picture of the patients’ variability of symptoms.

PwMS with more comorbid FNS showed poorer work ability than pwMS with more symptoms explained by structural pathology due to MS when measured by working hours per day (subjective measure) and by the reception of a disability pension (objective measure). Regarding the health-related quality of life, pwMS with more comorbid FNS had fewer limitations in physical activities than pwMS with more symptoms explained by structural pathology. As for other subscales, a higher comorbid FNS burden was associated with more limitations in social and usual daily activities at home and work due to emotional and physical problems including bodily pain. These patients also reported having poorer general health perceptions, worse mental health and more fatigue than pwMS with more symptoms explained by structural pathology. Almost all of the mentioned comparisons showed moderate to large effect sizes.

Our prevalence findings are consistent with previous research (3, 5) and show the importance of paying attention to FNS comorbidity with regard to diagnostics and therapy in structural neurological disorders such as MS. Even more so, considering that comorbid FNS in MS seem to have an adverse impact in terms of work ability and various domains of health-related quality of life despite having fewer limitations in physical activities. These results are consistent with previous findings related to the negative effects of FNS on health-related quality of life (15), mental health and work ability (14).

Ratings of the extent to which physical symptoms are explained by pathophysiological disorders showed to be useful in assessing functional somatic and neurological symptoms (3, 12, 13, 19–21). This assessment method was recognized to be reliable (12) and valid (13). We adapted the symptom rating that was described by Carson and Stone and used a five-point Likert scale instead of four answer options in order to make statistically more differentiated statements (3, 12, 13).

Dealing with the complex subject of comorbid FNS in structural neurological disorders, there are some limitations. One concern is that raters’ previous clinical experiences in evaluating comorbid FNS could affect rating the overall clinical picture and the symptoms. Moreover, neurologists may have more or different knowledge and experience in assessing “rule-in” examination signs for FNS than allied health practitioners. The results regarding sexual dysfunction should be interpreted with caution since neurologists rated symptoms reported in the last 7 days at the end of a patient’s stay and these symptoms could be under-reported due to the long stay at the clinic. Another limitation relates to the external validity which could be limited since the study was conducted in a setting of neurological rehabilitation. Including a broader spectrum of comorbid FNS that goes beyond the diagnostic criteria of DSM-5 might be considered a further limitation of this study. Despite carefully considering other reasons for subjective symptoms such as pain and fatigue we cannot exclude the possibility that some of these symptoms might be due to alternative causes and not directly related to MS or FNS. However, pain and cognitive disorders often comprise functional components (22, 23). In cognitive and motor fatigue, the distinction from fatigability is helpful in determining the extent of functional components (24). Furthermore, dissociative coping styles in pwMS seem to significantly increase fatigue (25). Including these symptoms can also be considered a strength of this study since it allows us to take into account the reality of everyday clinical practice where such symptoms are often present. In this sense, our study draws attention to clinically relevant issues that require further clarification. We did not evaluate the presence of comorbid psychiatric disorders and their possible association with FNS in our study, which could be considered a further shortcoming of this study. Future research should address this issue since psychiatric disorders such as anxiety and depression are very common in both MS (26) and functional neurological disorders (27) and could also impact HRQoL and work ability in pwMS with more comorbid FNS. Moreover, it seems important to take into account possible biological correlates of comorbid FNS in pwMS to establish possible pathophysiological mechanisms and offer better and more individualized treatment.

Our findings demonstrate that neurologists and allied health practitioners rated many pwMS as having neurological symptoms that could not be sufficiently explained by underlying structural pathology due to MS. More than half of our sample showed at least some FNS that were related to poorer health-related quality of life and lower work ability. Therefore, FNS pose an important comorbidity not only in regard to an individual burden but also in terms of health economics and societal costs. Altogether, our findings denote the importance of considering FNS in pwMS because of the high comorbidity rate and high functional interrelationship but also due to the socioeconomic relevance of such comorbidity.

In regard to therapy, treating functional neurological disorders in multidisciplinary inpatient settings has shown good outcomes (28–30) which is in line with our clinical experience at Kliniken Schmieder Konstanz in treating such patients (31, 32).

## Data availability statement

The raw data supporting the conclusions of this article will be made available by the authors, without undue reservation.

## Ethics statement

The studies involving human participants were reviewed and approved by Ethics Committee of the University of Konstanz. The patients/participants provided their written informed consent to participate in this study.

## Author contributions

KP, MD, CD, MJ, JL, and RS contributed to conception and design of the study. KP organized the database, collected data, and wrote the manuscript. MD and KP performed the statistical analysis. All authors contributed to the interpretation of data. All authors contributed to the article and approved the submitted version.

## Funding

This study was funded by Lurija Institute for Rehabilitation Sciences and Health Research, Allensbach.

## Conflict of interest

The authors declare that the research was conducted in the absence of any commercial or financial relationships that could be construed as a potential conflict of interest.

## Publisher’s note

All claims expressed in this article are solely those of the authors and do not necessarily represent those of their affiliated organizations, or those of the publisher, the editors and the reviewers. Any product that may be evaluated in this article, or claim that may be made by its manufacturer, is not guaranteed or endorsed by the publisher.
